# Caffeine Intake and Its Sex-Specific Association with General Anxiety: A Cross-Sectional Analysis among General Population Adults

**DOI:** 10.3390/nu14061242

**Published:** 2022-03-15

**Authors:** Indira Paz-Graniel, Junko Kose, Nancy Babio, Serge Hercberg, Pilar Galan, Mathilde Touvier, Jordi Salas-Salvadó, Valentina A. Andreeva

**Affiliations:** 1 Sorbonne Paris Nord University, Inserm U1153, Inrae U1125, Cnam, Nutritional Epidemiology Research Unit (EREN), Epidemiology and Statistics Research Center (CRESS), 74 Rue Marcel Cachin, 93017 Bobigny, France; indiradelsocorro.paz@urv.cat (I.P.-G.); j.kose@eren.smbh.univ-paris13.fr (J.K.); s.hercberg@eren.smbh.univ-paris13.fr (S.H.); p.galan@eren.smbh.univ-paris13.fr (P.G.); m.touvier@eren.smbh.univ-paris13.fr (M.T.); 2 Universitat Rovira i Virgili, Department of Biochemistry and Biotechnology, Human Nutrition Unit, 43201 Reus, Spain; nancy.babio@urv.cat (N.B.); jordi.salas@urv.cat (J.S.-S.); 3 Institut d′Investigació Sanitària Pere Virgili (IISPV), 43201 Reus, Spain; 4 Consorcio CIBER, M.P. Fisiopatología de la Obesidad y Nutrición (CIBERObn), Instituto de Salud Carlos III (ISCIII), 28029 Madrid, Spain; 5 Department of Public Health, AP-HP Avicenne Hospital, 93017 Bobigny, France

**Keywords:** caffeine, dietary intake, anxiety, STAI-T, mental health, epidemiological study

## Abstract

(1) Background: Caffeine is one of the most consumed psychoactive stimulants worldwide. It has been suggested that caffeine intake at large doses can induce anxiety, whereas evidence of the role of low to moderate caffeine intake is scarce and inconsistent. Therefore, we aimed to assess the association between caffeine intake and general anxiety in adults recruited from the general population. (2) Methods: Participants from the French NutriNet-Santé web cohort with data on caffeine intake and general anxiety (assessed during 2013–2016 through the trait subscale of Spielberger’s State-Trait Anxiety Inventory Form Y; STAI-T, sex-specific top quartile = high trait anxiety) were included in this cross-sectional analysis (n = 24,197; 74.1% women; mean age = 53.7 ± 13.9 years). Mean dietary intake was estimated using ≥2 self-reported 24-h dietary records. Sex-specific tertiles of caffeine intake and low/high trait anxiety were calculated. Multivariable logistic regression models were fitted to assess the odds ratio (OR) and 95% confidence interval (CI) for the association between caffeine intake and general anxiety by sex. (3) Results: In the total sample, the mean caffeine intake (mg/day) from all dietary sources combined was 220.6 ± 165.0 (women = 212.4 ± 159.6; men = 243.8 ± 177.7, *p* < 0.01). Women in the highest tertile of caffeine intake showed significantly higher odds for high trait anxiety compared to those in the lowest tertile (reference), even after adjustment for potential confounders (OR: 1.13; 95% CI: 1.03–1.23). No significant associations were detected among men. Sensitivity analyses according to perceived stress level and sugar intake, respectively, showed similar results. (4) Conclusions: The results suggest that higher caffeine intake is associated with higher odds of general anxiety among women but not among men. Further research is needed to confirm the sex-specific findings and elucidate the potential causal relationship between caffeine intake and anxiety status.

## 1. Introduction

In the last decade, mental health has been recognized as an important component of public health; it is characterized not only by the absence of mental disorders or disabilities but also by a general state of well-being, allowing the individual to conduct his or her daily life activities and to manage stressful situations [1]. The DSM-5 includes a wide range of mental conditions [2]; among them, anxiety disorders (along with depression) have been associated with the greatest disease burden [3]. They have been identified as one of the most common causes of the reduction in disability-adjusted life years, and have been associated with the risk of other mental and chronic physical conditions [3].

According to the World Health Organization (WHO), the prevalence of anxiety disorders, which are characterized by a sense of tension, apprehension, and especially fear, with an intensity that can range from mild to severe [2], has increased worldwide by 15% since 2005, and in 2015 nearly 264 million individuals had anxiety [4]. Moreover, it was recently reported that during the Coronavirus disease 2019 (COVID-19) pandemic, the global anxiety disorder prevalence increased by an estimated 25% [5], placing these conditions near the top of the list of urgent health concerns and highlighting the necessity of designing prevention strategies.

Anxiety disorder onset can be triggered by the interplay of several risk factors, such as adverse life experiences in early life, shyness, family history of mental illness, biological predispositions, environmental events, socio-economic disadvantage, heavy use of alcohol and/or illicit drugs, chronic medical conditions, etc. [6]. In addition, as a higher prevalence among women than men has been reported [4], it has been proposed that sex hormones might also play an important role in its etiology and chronicity [6].

As anxiety onset results from complex gene–environment interactions, prevention strategies should be especially focused on potentially modifiable factors, such as diet and physical activity. In this regard, it has been proposed that due to its ability to modulate the gut microbiome, inflammation, oxidative stress, and immune function, diet might play an important role in the prevention and management of anxiety [7,8]. Some evidence has suggested an inverse association between anxiety and adherence to healthy dietary patterns (characterized by a high content of fruit, vegetables, nuts, whole grains, and fish) [9], while a “Western diet” (with a high content of red meat, processed and/or fried food, simple sugars, and salt) has been positively associated with anxiety [10]. However, most research in this field has focused on dietary patterns, food consumption, or nutrient intake, while research about the relationship between beverage consumption and mental health has been scarce and mainly explored through the association with certain compounds such as alcohol and—to a lesser extent—caffeine [11,12]. Moreover, research on caffeine intake and its association with anxiety has been especially focused on the effect of very large doses (>200 mg per drinking occasion or >400 mg/day), and most of it has been conducted in susceptible individuals (i.e., those with specific psychopathological conditions, genetic predisposition, etc.) [12]. A handful of studies have reported that low doses of caffeine might produce stimulation, improve the performance of activities that require alertness, and decrease depression and even anxiety [13,14,15,16,17]. Nevertheless, evidence of the role of low to moderate doses of caffeine intake in healthy individuals or by sex is inconsistent and scarce [12,13,14]. As caffeine might be the most consumed psycho-stimulant around the world, and due to its likely complex effect on anxiety, further research in this field is needed. Moreover, results from recent studies suggested an increase in coffee, tea, and energy drink consumption during the COVID-19 pandemic [18,19,20]; as these beverages are the principal caffeine sources, caffeine intake might have also increased, and have become an additional risk factor for anxiety during this period and possibly beyond. The reported increased anxiety disorder prevalence during the COVID-19 pandemic in 2020–2021 underscores the urgency to strengthen mental illness prevention strategies.

In this context, the main objective of the present study was to explore the association between the usual intake of low–moderate caffeine doses and general anxiety in a large sample of adults recruited from the general population. We hypothesized that participants with a higher mean daily caffeine intake would present higher odds of anxiety. In addition, as women show a higher prevalence of anxiety disorders than do men, we further hypothesized that women would be more prone to show an unfavorable association between caffeine intake and anxiety.

## 2. Materials and Methods

### 2.1. Study Population

A cross-sectional analysis using data from the NutriNet-Santé web cohort was performed. Briefly, the NutriNet–Santé study is an ongoing web-based general population prospective cohort launched in France in 2009. It aims to elucidate the multifaceted association between nutrition and health, as well as the determinants of dietary behaviors and nutritional status. A detailed description of the NutriNet-Santé study has been published elsewhere [21]. It is registered at clinicaltrial.gov as NCT03335644 and was approved by the Institutional Review Board of the French Institute for Health and Medical Research (INSERM # 00000388FWA00005831) and by the National Commission on Informatics and Liberty (CNIL # 908,450 and # 909216).

Eligible participants are adults aged ≥18 years with internet access; recruitment relies on recurrent large multimedia campaigns. After providing electronic informed consent, the participants complete a set of five self-report questionnaires to assess sociodemographic and lifestyle characteristics, anthropometrics, dietary intake, physical activity, and health status (outlined below, and extensively described in prior publications [22,23,24,25,26,27]). The present analysis has been conducted with those participants who had data on caffeine intake and who had responded to the Spielberger’s State-Trait Anxiety Inventory Form Y (STAI-T) (both described below).

### 2.2. Assessment of Caffeine and Dietary Intake

At inclusion and every six months thereafter, participants are asked to complete three 24-h dietary records covering two weekdays and one weekend day. The tool used for collecting dietary data has been validated against dietitian interviews and nutritional status biomarkers [22,27]. Participants are asked to report all food and beverages consumed during the previous 24 h, considering the three main meals (breakfast, lunch, and dinner) and any other eating occasions. For the present analysis, we aimed to capture usual dietary intake, thus participants who had completed ≥ 2 dietary records within 2.5 years around the completion date of the STAI-T scale were eligible. All dietary data were weighted to account for weekday and weekend day consumption. Portion sizes were estimated using previously validated photographs [23] or usual containers. To calculate mean daily energy and nutrient intake, the NutriNet-Santé food composition database which includes > 3500 different items was used [28]. Participants with under-reported energy intake, identified via Black’s method [29] considering the participant’s age, sex, weight, height, physical activity level, and basal metabolic rate, were excluded from the analysis.

In the present study, caffeine intake (mg/day) was the main exposure variable. Total dietary caffeine consumption was estimated from 24-h dietary records [22] taking into consideration the average amount of caffeine contained in caffeinated coffee drinks (including Viennese coffee, Americano, espresso, mocha, Liège, gourmet, instant coffee, etc.), decaffeinated coffee, tea (including white, green, and black teas), regular soda, artificially sweetened soda, energy drinks, and alcohol-containing caffeinated beverages.

### 2.3. Trait Anxiety

Trait anxiety was assessed using the validated French version of the STAI-T scale [30], which was completed by participants only once between 2013 and 2016 as part of the NutriNet-Santé follow-up. Briefly, the STAI is one of the most widely used screening tools for assessing anxiety as a temporary state (STAI-S) and anxiety as a personality trait (STAI-T) reflecting general anxiety proneness [31]. In this study, trait anxiety was the outcome of interest, which is considered a relatively stable personal characteristic displayed in a wide range of daily life situations [31]. The STAI-T subscale consists of 20 items based on a 4-point Likert scale with responses ranging from “Almost never” to “Almost always.” The total score ranges from 20 to 80, with higher scores corresponding to higher levels of general anxiety symptoms [32]. As there is no established cut-off point for defining high trait anxiety, we first explored the value distribution and then applied the sex-specific top quartile as cut-off, which is consistent with prior studies [33,34]. In addition, as a higher prevalence of anxiety disorders in women compared to men has been reported [4], sex-specific analyses were conducted (described below).

### 2.4. Assessment of Covariates

At inclusion and yearly thereafter, participants provide self-reported information by completing validated questionnaires on sociodemographic characteristics and lifestyle [25], anthropometric measurements, and health status. Body mass index (BMI, kg/m^2^) was calculated based on self-reported height and weight [24]. Leisure-time physical activity was assessed through the short version of the International Physical Activity Questionnaire [26]. For each participant, we used covariate data obtained within a 2.5-year window around the date of STAI-T completion. As there is evidence that stress can trigger anxiety [6], we assessed whether the association between caffeine intake and trait anxiety might vary according to stress level. The validated French version of Cohen’s 10-item Perceived Stress Scale (PSS-10) was administered at the same time as the STAI-T. The PSS-10 is commonly used in epidemiological research; it assesses the degree to which situations in daily life were appraised as stressful during the previous month [35]; higher scores indicate higher perceived stress, without an established cut-off. In the total studied sample, the Pearson correlation coefficient between the PSS-10 and STAI-T was 0.74 (n = 24,197, *p* < 0.01), which served as further justification for the interaction tests and subgroup analyses (described below).

Participants with missing data on covariables were excluded, except for covariables with >5% missing values, in which case a “Missing data/not reported” category was created. Regarding the “socio-professional category” variable, if the value was missing and age was <25 or >60 years, the respective status of “student” and “retired” was attributed.

### 2.5. Statistical Analysis

Study participants were categorized into sex-specific tertiles of caffeine intake due to the value distribution of the caffeine intake variable and also for purposes of interpretability of the results. To compare study participants’ characteristics in terms of tertiles of caffeine intake, the x^2^ test and the ANOVA test were used, as appropriate. Participants in the highest quartile were considered as having high trait anxiety (i.e., STAI-T score = Q4 defined as “high trait anxiety” versus STAI-T < Q4 “low trait anxiety”). Interaction between caffeine intake tertiles and sex was tested including cross-product terms in crude and adjusted models (*p*-value for interaction < 0.01), considering the higher prevalence of anxiety disorders among women than among men. Given the significant results of these tests, the main analyses were stratified by sex. Interaction tests with age and smoking status with regard to caffeine intake were also performed but the results were not significant. Multivariable logistic regression models according to sex were fitted to assess the association (odds ratio (OR); 95% confidence interval (CI)) between tertiles of caffeine intake (lowest tertile as reference category) and the odds of high trait anxiety. Model 1 was adjusted for age (continuous scale). Model 2 was additionally adjusted for marital status (living alone, and married/cohabiting), educational level (less than high school, high school, college, and graduate), physical activity (low, moderate, and high), smoking status (never, former, and current smoker), socio-professional category (homemaker, manual work, professional, and retired), BMI (continuous scale, kg/m^2^), mean total energy intake (Kcal/d), alcohol consumption (continuous scale, g/dayay), and number of 24-h dietary records (continuous scale).

Sensitivity analyses by degree of perceived stress (PSS-10 score ≥ sex-specific mean) [36] were conducted to evaluate whether the associations observed in the main analysis were varied by perceived stress level. Further, we performed another set of sensitivity analyses, fitting statistical models where intakes of total sugar (g/day), simple sugars (g/day), and added sugars (g/day) were added as covariates to take into account the plausible association of these nutrients with anxiety [37] as well as the frequently combined intake of sugar and caffeine. The data were analyzed using the Stata 14 software (StataCorp, College Station, TX, USA), and statistical significance was set at a two-tailed *p*-value < 0.05.

## 3. Results

### 3.1. Sample Description

**Figure 1:**
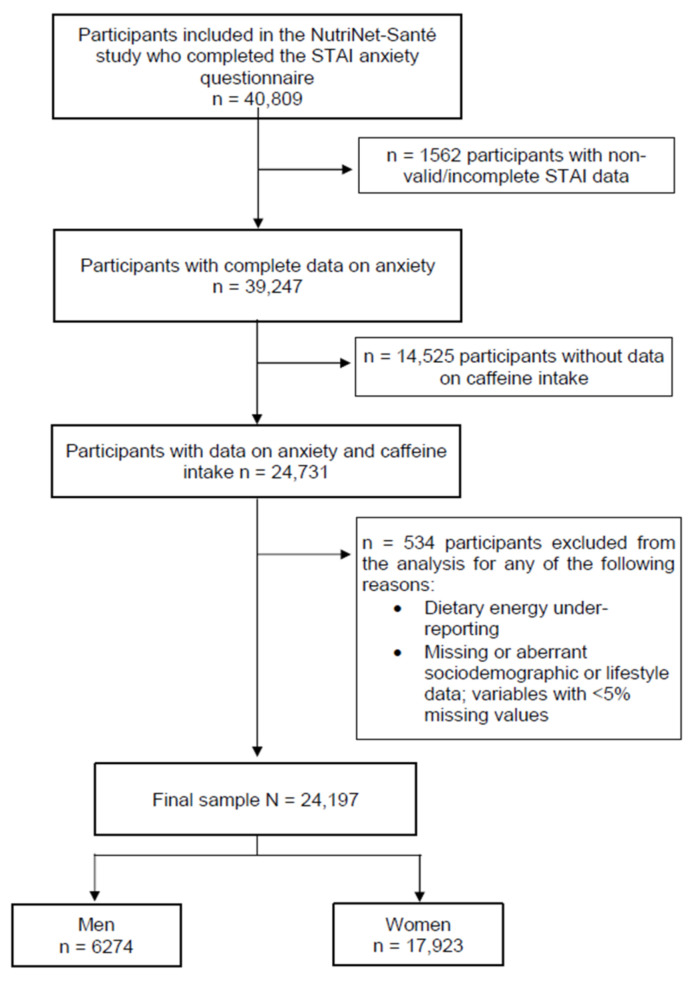
Participant flow diagram for NutriNet-Santé e-cohort participants included in the current analysis. Abbreviations: STAI-T, Spielberger’s State-Trait Anxiety Inventory Form Y.

From the 40,809 NutriNet-Santé participants who completed the anxiety questionnaire, we excluded from the present analysis individuals: (1) with non-valid or incomplete STAI-T data, (2) without data on caffeine intake, (3) with under-reported dietary intake or with < 2 24-h dietary records, and (4) with <5% missing data on sociodemographic and/or lifestyle variables. Therefore, the present analysis included 24,197 individuals (6274 men and 17,923 women) (Figure 1) with a mean age of 53.7 ± 13.9 years. In the full sample, the mean number of 24-h dietary records for the assessment period considered was 8.2 ± 3.8. The descriptive characteristics of the final sample according to sex-specific tertiles of caffeine intake are presented in Table 1. In the total studied population, the mean caffeine intake was 220.6 ± 165 mg/day. Significant differences by tertiles of caffeine intake were observed for all characteristics in men, except for educational level and perceived stress. Similarly, among women, except for high trait anxiety prevalence and perceived stress, all descriptive characteristics were significantly different across tertiles of caffeine intake (*p* < 0.05).

### 3.2. Description of Caffeine Intake

Table 2 shows the total daily consumption (mL/day) of various caffeinated beverages in the sample. In the total studied population, the mean total caffeinated beverages consumption was 385.0 ± 290.4 mL/day, and tea was the most consumed caffeine-containing beverage. Compared to men, women reported higher consumption of caffeinated beverages, especially tea. Caffeinated coffee was the most consumed caffeinated beverage in men.

### 3.3. Association between Caffeine Intake and Trait Anxiety

Table 3 summarizes the results of the sex-specific associations (OR, 95% CI) between tertiles of caffeine intake and the odds of high trait anxiety. No significant associations were observed for men in crude or adjusted models. In turn, women in the two higher tertiles of caffeine intake were more likely to present high trait anxiety (Model 2: aOR = 1.10, 95% CI: 1.01–1.20; aOR = 1.13 95% CI: 1.03–1.23, respectively) compared to those in the lowest tertile.

### 3.4. Sensitivity Analysis

As noted above, PSS-10 and STAI-T were significantly correlated (n = 24,197, *r* = 0.74), thus we proceeded with subgroup models. Results from the sensitivity analyses, stratified by perceived stress (Sensitivity analysis 1) are displayed in Table 4. Among women with high perceived stress, trait anxiety was positively associated with the highest tertile of caffeine intake (aOR = 1.17, 95% CI: 1.02–1.34), whereas no significant associations were observed for men. Results following the additional adjustment for total sugars, simple sugars, and added sugars (Sensitivity analysis 2, Table 5) remained virtually unchanged and at the same significance level as the main results.

## 4. Discussion

To the best of our knowledge, this large epidemiological study is the first to assess the association between caffeine intake and trait anxiety in a cohort recruited from the general adult population. The results of our analyses showed that among women caffeine intake was positively associated with trait anxiety, supporting our hypothesis. No significant associations were observed among men, however, this merits confirmation before a conclusion could be drawn. In our sample, women allocated in the highest tertile of caffeine intake, generally reported a 50 mg/day lower mean caffeine intake than did men in the top caffeine intake tertile, which is in line with data reported from two European cohorts [38].

It has been reported that anxiety prevalence is twice as high among women compared to men [4]. The increased odds of anxiety disorders in women compared to men have been partly attributed to differences in hormonal and neurotransmitter profiles (e.g., estrogen, progesterone, corticotropin-releasing factor, serotonin, etc.) [39]. Further, consistent with the evidence in the literature [40], in our study population, men had higher mean alcohol consumption than did women. It has been suggested that men are more likely than women to use substances with depressant effects on the central nervous system to cope with anxiety symptoms [41]. These factors might contribute to partly explain the different results among men and women. Interestingly, men and women also seem to differ regarding their motives for caffeine consumption [42].

Unlike our findings, some previous studies have reported significant associations between caffeine intake and anxiety in men; the discrepancies might be due to methodological differences (study design, individuals’ health status, age distribution, assessment tools, etc.). For example, in an experimental study (n = 99; 39 men, 60 women) conducted by Botella et al. [43], it was reported that after the consumption of coffee containing 150 mg or 300 mg of caffeine, men showed higher state anxiety than did women. In contrast, some authors have reported that low to moderate doses of caffeine reduced anxiety and increased mood in men [44]. In another experimental study conducted by Rogers et al., it was observed that at baseline higher habitual caffeine intake was associated with greater anxiety; however, after the intake of two caffeine doses (100 + 150 mg), participants who did not usually consume it showed increased anxiety [45]. In the latter three studies, samples did not include women [44] or results were not reported by sex [45], which does not allow comparisons with our observations. Overall, it should be noted that results from experimental studies largely pertain to the acute effect of caffeine intake in state anxiety but it has been weakly correlated with trait anxiety, which reflects a more habitual reaction to adverse events or stimulants [12,46]. In this regard, as caffeine intake can result in the development of tolerance, future studies of the association between caffeine and trait anxiety should be focused on long-term exposure. Moreover, caffeine intake at high doses (>400 mg/day, 200 mg per occasion) has been described as inducing anxiety disorders [2] but the effect of habitually lower doses remains unclear [47]. In turn, anxiogenic effects of caffeine have been more commonly reported in sensitive individuals, i.e., those with a genetic predisposition, younger age, with certain psychiatric disorders, and those with a predisposition to anxiety disorders than among their counterparts without such sensitivity profiles [12,48]. In our sample, largely composed of healthy French adults, mean caffeine intake (220.6 ± 165 mg/day) was somewhat lower than the quantities reported in previous studies in which caffeine intake was associated with anxiety [12,47]. This might serve as an additional explanation for the non-significant results in men. Caffeine intake in our sample was within the range of the reported usual consumption in the general French population (mean: 168 mg/day, P95: 438 mg/day) [49].

As stress has been associated with anxiety [6], we conducted sensitivity analyses by additionally stratifying the sample by level of perceived stress. The associations observed in the main analysis remained significant among women who reported having high perceived stress (PSS-10 score ≥ sex-specific mean); in men, perceived stress did not make a difference in the results. Stress is a biological response of the organism to situations perceived as threatening, in which the hypothalamic–pituitary–adrenocortical axis and the sympathetic–adrenal–medullary axis are activated, leading to stress hormone release (cortisol and catecholamines) [50]. Moreover differences in the biological and psychological stress response by sex have been described. It has been suggested that women are more susceptible to stress-induced anxiety than men, especially due to lower basal stress hormone levels in women, in addition to the role of sex hormones involved in the stress response [50,51,52]. It has also been suggested that women and men react to stressful life situations differently; while women might be more likely to ruminate (through disturbing and repetitive thoughts that are distressing and debilitating, and consequently might have increased anxiety symptoms), men tend to engage in more active pathways for problem-solving (i.e., behavioral and cognitive strategies focused on changing the stressful condition, and its perception and meaning), problem avoidance, and social withdrawal [41] that may increase the risk for stress-induced anxiety. Next, we previously reported an association between intakes of added sugars (g/day) and trait anxiety [37]. As caffeine is frequently consumed along with sugar, and to take into consideration the previously observed plausible associations, a second set of sensitivity analyses was performed by additionally adjusting our models for total sugar (g/day), simple sugars (g/day), and added sugars (g/day), and found that the results remained virtually unchanged. This gives greater robustness to our results about the potential effect of caffeine intake on trait anxiety.

As previous studies have reported a positive association between smoking habits and coffee consumption [53], and increased caffeine metabolism in smokers has been suggested [38], we tested the interaction between smoking status and caffeine intake, yet the result was non-significant. In our sample, men did not seem to be heavier smokers than women; that might partially explain this result. In addition, previous evidence has suggested a weaker association between smoking habits and tea consumption, in this regard, the differences observed in caffeinated beverage (tea and coffee) consumption among the sexes might be one reason for the lack of significant interactions. Future specific analyses are needed to clarify these potential interactions and their relationship with anxiety.

Several mechanisms underlying the observed associations between caffeine intake and general anxiety have been suggested in the literature. It has been proposed that the caffeine effect might be mediated via (a) the antagonism of adenosine receptors, (b) the inhibition of phosphodiesterase, (c) intracellular stores/calcium release, and (d) the antagonism of GABA_A_ receptors [54]. Due to its similarity to adenosine, the most accepted mechanism regarding the impact of caffeine is its antagonistic property at the level of adenosine receptors. It has been proposed that caffeine might bind to adenosine receptors and promote behavioral alertness, such as vigilance, attention, elevated mood, and arousal [55]. Meanwhile, phosphodiesterase inhibition and calcium release mechanisms require very high doses (>25 mg/kg/day) of caffeine to occur, and therefore, the pathway of their impact is less pertinent [54]. Next, some polymorphisms of A2A adenosine receptors have been described which may explain discrepancies in findings among different populations [12]. Finally, the source of caffeine might play an important role in its effect on trait anxiety. In our study, men were more likely to consume coffee than women. Coffee, due to its complex matrix featuring vitamins, minerals, bioactive phytochemicals, and polyphenols with antioxidant properties [56], might present a synergistic interaction of caffeine with the other components, thus potentially promoting a protective effect against anxiety. Further, tea has usually been associated with relaxation states, however, a cup of tea provides approximately 35–61 mg of caffeine and 4.5–22.5 mg of theanine [57], and therefore its consumption might promote anxiety symptoms, especially when such consumption is increased. Nevertheless, in an experimental analysis conducted by Smith et al., the effect of caffeine on mood and performance was not modified by the type of drink from which it was obtained [14]. Future studies should explore the potential interaction among the various beverage compounds and their individual and combined effects on mood and anxiety.

Our study has certain limitations that must be considered. One of the main limitations is the potential reverse causation bias, inherent in the cross-sectional design; in addition it is not possible to determine a causal relationship between caffeine intake and general anxiety. Second, despite the STAI-T being one of the most commonly used assessment tools regarding general anxiety proneness in epidemiological research, it cannot substitute for a complete clinical diagnosis. Third, caffeine intake was estimated from dietary sources and no other potential sources (such as dietary supplements) were considered; it is, therefore, possible that mean caffeine intake might have been underestimated. Still, as noted above, the reported mean caffeine intake in our studied population was within the range of the reported usual consumption in the general French population [49]. Fourth, our study used data from the NutriNet-Santé cohort, in which women and well-educated individuals are somewhat over-represented in comparison with the general French population [58], therefore the findings should be extrapolated prudently. Finally, we cannot discount the possibility that the observed associations might be due to residual confounding by factors not taken into account in the statistical analysis (clinical and family history of anxiety disorders, ethnoracial status, etc.). As study strengths, however, it should be highlighted that the analysis was based on a large and heterogeneous sample and data were obtained via validated assessment tools. Moreover, dietary (and caffeine) intake was estimated using a large number of previously validated 24-h dietary records [22], reducing the risk of over- or under-estimation.

## 5. Conclusions

In this large cross-sectional study conducted in a sample of adults recruited from the French general population, higher caffeine intake was positively associated with higher odds of general anxiety in women but not in men. Further research is needed to confirm the sex-specific findings and to elucidate the potential mechanisms involved in the observed association. The findings could inform future mental health promotion initiatives, especially among women.

## Figures and Tables

**Table 1:** Descriptive characteristics of the NutriNet-Santé participants according to sex and tertiles of caffeine intake.

	Full Sample	Men				Women			
	n = 24,197	T1 n = 2092	T2 n = 2091	T3 n = 2091	*p* Value ^a^	T1 n = 5975	T2 n = 5974	T3 n = 5974	*p* Value ^b^
Caffeine intake, mg/day	220.6 ± 165	76.0 ± 47.7	218.8 ± 38.5	436.8 ± 157.8	<0.01	62.3 ± 36.8	186.4 ± 35.8	388.6 ± 139.5	<0.01
High trait anxiety ^†^		23.4 (489)	20.7 (433)	20.4 (427)	0.03	23.8 (1419)	24.1 (1438)	24.4 (1459)	0.69
Age, mean (SD), years	53.7 ± 13.9	57.1 ± 14.8	60.8 ± 12.4	59.1 ± 12.0	<0.01	47.8 ± 14.7	53.5 ± 13.3	54.3 ± 11.9	<0.01
Age category					<0.01				<0.01
<40 years	20.7 (5019)	17.7 (370)	8.8 (184)	8.9 (185)		36.9 (2204)	19.9 (1191)	14.8 (885)	
40–60 years	39.1 (9454)	28.4 (595)	27.7 (579)	34.9 (730)		36.5 (2179)	42.1 (2515)	47.8 (2856)	
>60 years	40.2 (9724)	53.9 (1127)	63.5 (1328)	56.2 (1176)		26.6 (1592)	38.0 (2268)	37.4 (2233)	
Educational level					0.51				<0.01
Less than high school	13.9 (3372)	16.3 (340)	18.4 (384)	18.4 (386)		11.5 (686)	13.7 (818)	12.7 (758)	
High school or equivalent	16.8 (4058)	18.6 (389)	17.4 (364)	17.4 (364)		16.0 (955)	16.6 (992)	16.6 (994)	
College, undergraduate degree	27.9 (6746)	22.9 (479)	21.9 (458)	22.6 (472)		30.9 (1843)	29.2 (1746)	29.3 (1748)	
Graduate degree	36.3 (8788)	40.0 (837)	39.4 (825)	39.2 (819)		36.7 (2194)	34.1 (2,037)	34.8 (2076)	
Not reported	5.10 (1233)	2.2 (47)	2.9 (60)	2.4 (50)		5.0 (297)	6.4 (381)	6.7 (398)	
Socio-professional category					<0.01				<0.01
Homemaker/disabled/unemployed/student	8.30 (2016)	4.3 (91)	2.2 (46)	3.4 (70)		11.5 (685)	9.3 (555)	9.5 (569)	
Manual/office work/administrative staff	30.5 (7389)	20.8 (436)	14.5 (304)	18.6 (389)		40.0 (2390)	32.1 (1920)	32.6 (1950)	
Professional/executive staff	23.1 (5578)	22.7 (475)	21.8 (4555)	24.5 (513)		23.4 (1398)	22.7 (1357)	23.1 (1380)	
Retired	38.10 (9214)	52.1 (1090)	61.5 (1286)	53.5 (1119)		25.1 (1502)	35.9 (2142)	34.7 (2075)	
Marital status					<0.01				<0.01
Living alone (single, divorced, widowed)	23.4 (5674)	20.5 (428)	14.8 (310)	14.9 (311)		24.6 (1470)	24.6 (1472)	28.2 (1683)	
Married/cohabiting	76.6 (18,523)	79.5 (1664)	85.2 (1781)	85.1 (1780)		75.4 (4505)	75.4 (4502)	71.8 (4291)	
Physical activity *					<0.01				<0.01
Low	36.8 (8906)	44.6 (932)	48.6 (1,107)	45.2 (944)		30.4 (1814)	34.5 (2058)	35.8 (2141)	
Moderate	41.3 (9986)	34.9 (730)	35.5 (742)	36.1 (755)		43.9 (2620)	43.3 (2588)	42.7 (2551)	
High	21.9 (5305)	20.5 (430)	15.9 (332)	18.7 (392)		25.8 (1541)	22.2 (1328)	21.5 (1282)	
Smoking status					<0.01				<0.01
Never smoker	51.0 (12,351)	52.7 (1103)	39.3 (821)	33.4 (699)		66.1 (3949)	52.6 (3142)	44.1 (2637)	
Former smoker	39.4 (9519)	41.9 (877)	53.0 (1110)	54.4 (1138)		27.3 (1629)	38.0 (2267)	41.8 (2498)	
Current smoker	9.6 (2327)	5.4 (112)	7.7 (160)	12.2 (254)		6.6 (397)	9.5 (565)	14.0 (839)	
Body Mass Index (BMI), kg/m^2^	23.8 ± 4.1	24.5 ± 3.5	25.0 ± 3.5	25.4 ± 3.6	<0.01	23.1 ± 4.2	23.4 ± 4.2	23.5 ± 4.3	<0.01
BMI category					<0.01				<0.01
Underweight (<18.5)	4.6 (1123)	1.7 (36)	0.9 (19)	0.6 (13)		6.7 (398)	5.7 (338)	5.3 (319)	
Normal weight (18.5–24.9)	64.4 (15,588)	59.7 (1250)	56.1 (1173)	50.1 (1048)		68.8 (4108)	67.9 (4056)	66.2 (3953)	
Overweight (25.0–29.9)	23.6 (5699)	32.3 (675)	35.7 (746)	39.8 (33)		17.5 (1048)	19.3 (1154)	20.8 (1243)	
Obese (≥30)	7.4 (1787)	6.3 (131)	7.3 (153)	9.4 (197		7.0 (421)	7.1 (426)	7.7 (459)	
Total energy intake, kcal/d	1910.8 ± 440.4	2223.1 ± 452.5	2286.1 ± 454.0	2,324.3 ± 463.3	<0.01	1759.8 ± 356.5	1772.5 ± 344.6	1814.8 ± 355.7	<0.01
Alcohol consumption, g ethanol/d	8.5 ± 11.6	12.4 ± 16.3	16.6 ± 16.0	16.8 ± 15.4	<0.01	4.5 ± 7.4	6.6 ± 8.3	7.5 ± 9.4	<0.01
Number of 24-h dietary records	8.2 ± 3.8	8.5 ± 3.7	9.0 ± 3.6	8.8 ± 3.7	<0.01	7.5 ± 3.8	8.1 ± 3.7	8.3 ± 3.7	<0.01
Perceived stress score^‡^	13.5 ± 6.9	11.6 ± 6.3	11.2 ± 6.2	11.3 ± 6.5	0.21	14.3 ± 6.9	14.2 ± 6.8	14.2 ± 6.9	0.48

Abbreviations: SD, standard deviation. Data expressed as percentage (number) or mean ± standard deviation, as appropriate. *p*-values for comparisons between tertiles of caffeine intake for men ^a^ and women ^b^ were calculated by Pearson’s chi-square test for categorical variables or one-factor ANOVA for continuous variables. ^†^ Spielberger Trait Anxiety Inventory (STAI-T) Form Y; score range between 20 and 80 points, with higher scores indicating higher anxiety symptomatology, high trait anxiety STAI–T score in Q4. * Assessed with the International Physical Activity Questionnaire-Short Form; scoring followed established protocol. ^‡^ Assessed with Cohen’s Perceived Stress Scale-10 (PSS-10) where higher scores indicate higher levels of perceived stress.

**Table 2:** Total daily consumption (mL/day) of various caffeinated beverages in the NutriNet-Santé study (n = 24,197).

Caffeine Sources	Total Sample	Men (n = 6274)	Women (n = 17,923)	*p* Value ^a^
Total caffeinated beverages	385.0 ± 290.4	343.8 ± 259.7	399.5 ± 299.1	<0.01
Total coffee	160.5 ± 179.9	188.3 ± 188.9	150.7 ± 175.6	<0.01
Caffeinated coffee	152.2 ± 174.9	179.9 ± 185.3	142.5 ± 170.1	<0.01
Decaffeinated coffee	8.2 ± 46.1	8.4 ± 47.9	8.2 ± 45.5	0.73
Tea	211.7 ± 280.5	143.0 ± 229.2	235.7 ± 292.6	<0.01
Other caffeinated beverages ^b^	12.9 ± 63.3	12.5 ± 53.4	13.0 ± 66.4	0.54

Data expressed as mean ± standard deviation. ^a^ *p*-value for comparisons between sexes was obtained by Student’s *t*-test. ^b^ Other caffeinated beverages category includes sodas, energy drinks, and alcohol-containing caffeinated beverages.

**Table 3:** Sex-specific associations (odds ratios, 95% CI) between tertiles of caffeine intake and odds of high trait anxiety in the NutriNet-Santé study (n = 24,197).

	Men			Women		
	T1 n = 2092	T2 n = 2091	T3 n = 2091	T1 n = 5975	T2 n = 5974	T3 n = 5974
Caffeine intake, mg/day ^	76.0 ± 47.7	218.8 ± 38.5	436.8 ± 157.8	62.3 ± 36.8	186.4 ± 35.8	388.6 ± 139.5
Trait anxiety ^†^ (% (n) high) ^	23.4 (489)	20.7 (433)	20.4 (427)	26.3 (1572)	26.2 (1566)	26.7 (1597)
Model 1	1 (ref.)	0.94 (0.81–1.08)	0.89 (0.76–1.03)	1 (ref.)	1.08 (0.99–1.18)	**1.11 (1.02–1.21)**
Model 2	1 (ref.)	0.96 (0.83–1.12)	0.88 (0.75–1.02)	1 (ref.)	**1.10 (1.01–1.20)**	**1.13 (1.03–1.23)**

Abbreviations: ref, reference category. ^ Data expressed as percentage (number) or mean ± standard deviation. Multivariable logistic regression models were fitted: outcome: STAI-T < Q4 (0) vs. STAI-T score in Q4 (1) ^†^; reference category: lowest tertile of caffeine intake. Model 1 adjusted for age. Model 2 additionally adjusted for marital status, education, physical activity, smoking status, socio-professional category, BMI, mean total energy intake, alcohol consumption, and number of 24-h dietary records. Bold means highlight significant results.

**Table 4:** Sensitivity analyses of the association (odds ratio, 95% CI) between tertiles of caffeine intake and odds of high trait anxiety according to perceived stress level in women and men from the NutriNet-Santé cohort.

	Women (n = 12,923)					
	Low Perceived Stress (PSS-10 Score < Sex-Specific Mean)			High Perceived Stress (PSS-10 Score ≥ Sex-Specific Mean)		
	T1 n = 3216	T2 n = 3216	T3 n = 3216	T1 n = 2759	T2 n = 2758	T3 n = 2758
Caffeine intake, mg/day ^	62.4 ± 37.3	186.9 ± 35.7	388.3 ± 135.1	62.2 ± 36.2	185.8 ± 36.0	389.0 ± 144.6
Trait anxiety ^†^ (% (n) high) ^	24.6 (790)	23.6 (759)	25.0 (805)	20.8 (574)	22.6 (623)	23.4 (646)
Model S3	1 (ref.)	0.96 (0.86–1.08)	1.05 (0.93–1.18)	1 (ref.)	1.14 (0.99–1.30)	**1.17 (1.02–1.34)**
	**Men (n = 6274)**					
	Low Perceived Stress (PSS-10 Score < Sex-Specific Mean)			High Perceived Stress (PSS-10 Score ≥ Sex-Specific Mean)		
	T1 n = 1114	T2 n = 1113	T3 n = 1113	T1 n = 978	T2 n = 978	T3 n = 978
Caffeine intake, mg/day^	78.7 ± 48.8	221.2 ± 37.2	435.8 ± 155.5	73.0 ± 46.4	215.9 ± 39.9	437.9 ± 160.5
Trait anxiety ^†^ (% (n) high)^	23.9 (267)	24.8 (276)	23.9 (266)	24.3 (238)	25.2 (246)	24.3 (238)
Model S3	1 (ref.)	1.09 (0.89–1.33)	1.02 (0.83–1.24)	1 (ref.)	1.17 (0.95–1.45)	1.04 (0.84–1.30)

Abbreviations: ref, reference category. ^ Data expressed as percentage (number) or mean ± standard deviation. Multivariable logistic regression models were fitted: outcome: STAI-T < Q4 (0) vs. STAI-T score in Q4 (1) ^†^; reference category: lowest tertile of caffeine intake. All models were adjusted for age, marital status, education, physical activity, smoking status, socio-professional category, BMI, mean total energy intake, alcohol consumption, and number of 24-h dietary records. Bold means highlight significant results.

**Table 5:** Sensitivity analyses of the association (odds ratio, 95% CI) between tertiles of caffeine intake and odds of high trait anxiety in women and men from the NutriNet-Santé cohort (n = 12,923) according to sugar intake.

	Men			Women		
	T1 n = 2092	T2 n = 2091	T3 n = 2091	T1 n = 5975	T2 n = 5974	T3 n = 5974
Caffeine intake, mg/day ^	76.0 ± 47.7	218.8 ± 38.5	436.8 ± 157.8	62.3 ± 36.8	186.4 ± 35.8	388.6 ± 139.5
Trait anxiety ^†^ (% (n) high) ^	23.4 (489)	20.7 (433)	20.4 (427)	26.3 (1572)	26.2 (1566)	26.7 (1597)
Model S1	1 (ref.)	1.01 (0.85–1.21)	0.90 (0.75–1.07)	1 (ref.)	**1.10 (1.01–1.20)**	**1.13 (1.03–1.23)**
Model S2	1 (ref.)	1.00 (0.85–1.21)	0.89 (0.75–1.07)	1 (ref.)	**1.10 (1.01–1.20)**	**1.13 (1.03–1.23)**
Model S3	1 (ref.)	1.01 (0.85–1.21)	0.89 (0.75–1.07)	1 (ref.)	**1.10 (1.01–1.20)**	**1.13 (1.03–1.23)**

Abbreviations: ref, reference category. ^ Data expressed as percentage (number) or mean ± standard deviation. Multivariable logistic regression models were fitted: outcome: STAI-T < Q4 (0) vs. STAI-T score in Q4 (1) ^†^; reference category: lowest tertile of caffeine intake. All models were adjusted for age, marital status, education, physical activity, smoking status, socio-professional category, BMI, mean total energy intake, alcohol consumption, and number of 24-h dietary records. Model S1 additionally adjusted for total sugars (g/day). Model S2 additionally adjusted for simple sugars (g/day). Model S3 additionally adjusted for added sugars (g/day). Bold means highlight significant results.

## Data Availability

Researchers at public institutions can submit a project collaboration request that includes information about their institution and a brief description of the project to: collaboration@etude-nutrinet-sante.fr. All requests are reviewed by the steering committee of the NutriNet-Santé study. In case of approval, a signed data access agreement will be requested and additional authorizations from the competent administrative authorities may be needed regarding human subjects’ data protection. In accordance with existing regulations, no personally identifiable data will be made available.
